# Correction of narrow nostril deformity secondary to cleft lip: indications for different surgical methods and a retrospective study

**DOI:** 10.3389/fped.2023.1156275

**Published:** 2023-05-03

**Authors:** Hongpu Wei, Xiaofeng Xu, Teng Wan, Yusheng Yang, Yong Zhang, Yilai Wu, Yun Liang

**Affiliations:** ^1^ Department of Oral and Craniomaxillofacial Surgery, Shanghai Ninth People's Hospital, Shanghai Jiao Tong University School of Medicine, Shanghai, China; ^2^ College of Stomatology, Shanghai Jiao Tong University, Shanghai, China; ^3^ National Center for Stomatology, Shanghai, China; ^4^ National Clinical Research Center for Oral Diseases, Shanghai, China; ^5^ Shanghai Key Laboratory of Stomatology, Shanghai, China; ^6^ Shanghai Research Institute of Stomatology, Shanghai, China

**Keywords:** cleft lip, narrow nostril deformities, diagnosis, rhinoplasty, surgical method selection

## Abstract

**Background:**

Cleft lip and/or palate (CLP) can lead to severe nasolabial deformities that significantly affect the appearance of the patient. Among all types of nasolabial deformities, narrow nostril deformities are the most troublesome, causing poor and unstable surgical outcomes. The purpose of this study was to develop an algorithm for surgical method selection for revision of narrow nostril deformities secondary to CLP based on retrospective clinical data.

**Materials and methods:**

Patients with narrow nostril deformities secondary to CLP were enrolled in the study. Before surgery, patients' clinical data were collected and the width of the nasal floor and the length of the alar rim were measured. Surgical methods were determined according to the measurements. After surgery, a nostril retainer was applied for 6 months to consolidate and maintain the nostril shape. The surgical method and postsurgical changes were recorded for the final summary of the algorithm to select surgical methods for narrow nostril deformities.

**Results:**

The data from 9 patients were analyzed. According to the width of the nasal floor and the length of the alar rim, correct surgical methods were determined. Four patients received nasolabial skin flaps to widen the soft tissue of the nasal floor. Three patients received upper lip scar tissue flaps to treat the narrow nasal floor. For the short alar rim, free alar composite tissue flap or narrowing of the nostril of the noncleft side was recommended.

**Conclusion:**

The width of the nasal floor and the length of the alar rim are critical elements to consider when selecting the correct surgical method for revising narrow nostril deformities secondary to CLP. The proposed algorithm provides a reference for selecting surgical methods in future clinical practice.

## 1. Introduction

Cleft lip and/or palate (CLP) is the most common birth defect in the world (1, 2). CLP causes morbidity to patients and imposes financial risks for families. With the popularization of prenatal diagnosis, the incidence of CLP in newborns is decreasing (3, 4). However, improvements in living standards have gradually increased people's aesthetic requirements. Secondary facial deformities after CLP include nasal and labial deformities. Although clefts and deformities are revised in the first stage of surgery, differences in growth rates still exist in bilateral tissues during the growth period (5, 6). The existence of scars and the asymmetry of the lip and nose can distress patients when interacting with other people (7). Thus, secondary rhinocheiloplasty performed in adulthood is regarded as the last surgery in a cleft patient's management timeline (8).

Nasal deformities secondary to CLP include asymmetry of the nostrils, deviations of the septum, and collapse of the nasal tip (8). Nostril asymmetry is the most common deformity in patients with unilateral cleft lip. The typical clinical characteristics of nostril asymmetry include collapse of the nostril on the cleft side combined with an increased base width and decreased height (9). However, the cleft side nostril is sometimes narrow; this type of nostril asymmetry is rare. The main cases of narrow nostril deformity secondary to cleft lip may include the following aspects. Firstly, the improper surgical correction to remove excessive tissue may be dominant reason for it. Besides that, irregular applying of nasal retainer after the primary open rhinoplasty and postoperative scar contracture may also contribute to the occurrence of deformity (5, 6, 9).

Few studies have focused on the causes and treatments of narrow nostril deformities secondary to CLP. Thus, the surgical results for this deformity are often unsatisfactory. In this study, we summarize the causes of narrow nostril deformities secondary to CLP and propose corresponding surgical techniques.

## 2. Materials and methods

From February 2010 to July 2021, 9 consecutive patients with narrow nostril deformities secondary to CLP who underwent surgical treatment at the Department of Oral and Craniomaxillofacial Surgery, Shanghai Ninth People's Hospital were enrolled in this retrospective case series. Informed consent was obtained from all study subjects before participating in the study. The protocol of this retrospective case series was approved by the institutional ethics committee of Shanghai Ninth People's Hospital.

Inclusion criteria were: ①Patients with narrow nostril deformity secondary to unilateral clefts lip and palate. The narrow nostril deformity was defined as the differences of bilateral nasal floor or alar rim was more than 2 millimeters. ②According to the requirements of patients and their families. All patients underwent open rhinoplasty to revise the narrow nostril deformity. ③Considering that the growth and development of the nose are basically completed by the age of 16, the minimum age for revision surgery of narrow nostril deformity was set as the age of 16. ④Patients had adequate and complete presurgical and postsurgical follow-up clinical records for at least 12 months. Exclusion criteria were a history of nasal trauma and incomplete medical records.

Surgeons fully communicated with the patient before surgery. Complete clinical data, including previous surgical history and facial photos, were collected. The surgery was performed under general anesthesia. After disinfecting, presurgical photos were taken and the presurgical deformity was measured before the surgery. To quantitatively measure the degree of deformity, we use a syringe needle to dip with methylene blue to locate the landmark point. The landmark point we used include bilateral lip peak point, philtrum point and bilateral cheilion point. By penetrating the skin with a syringe, the methylene blue is then stained in the subcutaneous tissue. In this way, we can locate landmark point and perform corresponding measurements. The alar rim was encircled with a steel wire and the length of the steel wire was measured to determine the length of the alar rim. The width of the nasal floor and the breadth of the vermilion and scar were measured using castroviejo calipers. After evaluating the degree of the deformity, various surgical methods were performed to correct the nasal and labial deformities secondary to CLP.

Two days after surgery, a nostril retainer was placed to consolidate and maintain the nostril shape. Patients were instructed to remove and clean the nostril retainer after 6 consecutive hours. Seven days after the surgery, sutures were removed and patients were instructed to apply the nostril retainer for 6 months. Postsurgical photographs were taken immediately, 1 month, 3 months, and 6 months after the surgery.

In all these patients, the surgical revision methods and the presence of postsurgical complications, such as infection and deformity recurrence, were monitored. During the follow-up, patient satisfaction and treatment outcomes were recorded and evaluated.

## 3. Results

Nine patients with narrow nostril deformities secondary to unilateral CLP were enrolled in the study. No recurrence of deformity occurred post surgically in all patients. And all patients were satisfied with the postsurgical results. Characteristics of the patient population, patient diagnosis, and surgical methods are summarized in Table 1. Since all these patients' primary surgeries had been performed for more than 15 years and were not performed in our center, it is not possible to obtain the surgical procedures and specific surgical technique of the primary surgery at this time. The mean age of the patients was 20.67 years (range 17–30 years, median 21 years). The mean age of the primary cheiloplasty was 5.89 months (range 3–12 months, median 6 months). The male: female ratio was 2:7. Five patients had left nasal deformities secondary to CLP and four patients had right nasal deformities secondary to CLP. Seven patients had unilateral cleft lip, cleft palate, and alveolar cleft; the other two patients only had unilateral cleft lip and cleft palate.

**Table 1:** Demographics, diagnosis, patient features and final surgical methods.

Patient No	Age	Gender	Age of cheiloplasty	Diagnosis	Combined cleft palate and alveolar cleft or not	Surgical time of rhinoplasty	Surgical methods of rhinoplasty
**1**	17	Male	8 months	L. Nasal deformity secondary to CLP	Cleft lip + cleft palate + alveolar cleft	August 10, 2013	Nasolabial skin flap
**2**	22	Female	4 months	R. Nasal deformity secondary to CLP	Cleft lip + alveolar cleft	June 22, 2010	Nasolabial skin flap
**3**	18	Female	3 months	R. Nasal deformity secondary to CLP	Cleft lip + cleft palate + alveolar cleft	July 23, 2013	Nasolabial skin flap
**4**	21	Female	7 months	L. Nasal deformity secondary to CLP	Cleft lip + cleft palate + alveolar cleft	May 8, 2014	Nasolabial skin flap
**5**	30	Female	4 months	L. Nasal deformity secondary to CLP	cleft lip + alveolar cleft	March 18, 2014	Upper lip scar tissue flap
**6**	17	Female	6 months	L. Nasal deformity secondary to CLP	Cleft lip + cleft palate + alveolar cleft	August 7, 2012	Upper lip scar tissue flap
**7**	22	Male	1 year	R. Nasal deformity secondary to CLP	Cleft lip + cleft palate + alveolar cleft	March 2, 2010	Upper lip scar tissue flap
**8**	22	Female	3 months	R. Nasal deformity secondary to CLP	Cleft lip + cleft palate + alveolar cleft	July 18, 2011	Free alar composite tissue flap + narrow the nostril of non-cleft side
**9**	17	Female	6 months	L. Nasal deformity secondary to CLP	Cleft lip + cleft palate + alveolar cleft	April 9, 2021	narrow the nostril of non-cleft side

Based on a specific summarization of this group of cases, we further classify the narrow nostril deformity into three main categories according to the morphological differences of bilateral nostrils. The first type is the narrow nostril deformity caused by narrow nasal floor, which characterized by the difference of bilateral nasal floor greater than 2 mm, while the alar rim of the affected side is normal. The second one is the narrow nostril deformity caused by short alar rim. Such patients are with extremely short alar rim and the difference of bilateral alar rim is greater than 2 mm, while the nasal floor of the affected side is normal. The third type is the combination of the two-above type. Such patients are characterized by having both a narrow nasal floor and short alar rim at the same time. The deformity of this type is troublesome and requires a combination of various methods to revise the severe deformity.

### 3.1. Clinical presentation 1

A 17-year-old female patient was referred to our department for the management of left nasal and labial deformities secondary to CLP. This patient underwent cheiloplasty at the age of 6 months at a local hospital. She received palatoplasty at the age of 6 years in our department. Repair of the left alveolar cleft with iliac bone harvest was conducted at the age of 12 years at another hospital. As the child grew, the nasal and labial tissue grew and changed. The repaired lip was affected and the deformity become more apparent. Thus, the patient was referred to our department to revise the nasal and labial deformities. Physical examination revealed a wide postoperative scar on the left upper lip. The left vermilion was hypertrophic, the left lip peak was raised, and the lip bread structure did not show. The left nasal tip was collapsed, and the nostril sizes were asymmetric. The asymmetry of bilateral nostrils was particularly evident in this patient from the bottom view. The surgical objectives included excision of the residual scar, repositioning of key anatomic landmarks, including the vermilion-cutaneous junction, leveling of the vertical lip lengths, enlargement of the nostril on the affected side, and repositioning of the alar cartilage. Before surgery, the width of the nasal floor and the length of the alar rim were measured to confirm the causes of the narrow nostril and establish a reasonable treatment strategy. The main cause of the narrowed nostril was a narrow nasal floor, according to the presurgical measurements. Thus, the wide residual scar was not completely resected during surgery, and the residual pedicle scar tissue flap was transposed and transplanted to repair the narrow nasal floor. This surgical technique would resect the scar on the upper lip, adjust the shape of the upper lip, broaden the nasal floor tissue, and resolve the narrow nasal floor at the same time. Besides that, to solve the problem of nasal alar lateral malposition, open rhinoplasty was performed meanwhile to reposition the nasal alar cartilage. During the surgery, the bilateral nasal alar cartilage was freely dissociated and anatomically reduced through the bilateral tajima incision and the inverted V-shaped incision on the nasal columella. The patient was instructed to apply the nostril retainer for 6 months after the surgery to reposition the alar cartilage and maintain the nostril size. After the secondary rhinocheiloplasty, a good nasolabial profile was acquired. The photos before and after the surgery and the schematic diagram of the surgery are shown in Figure 1.

**Figure 1:**
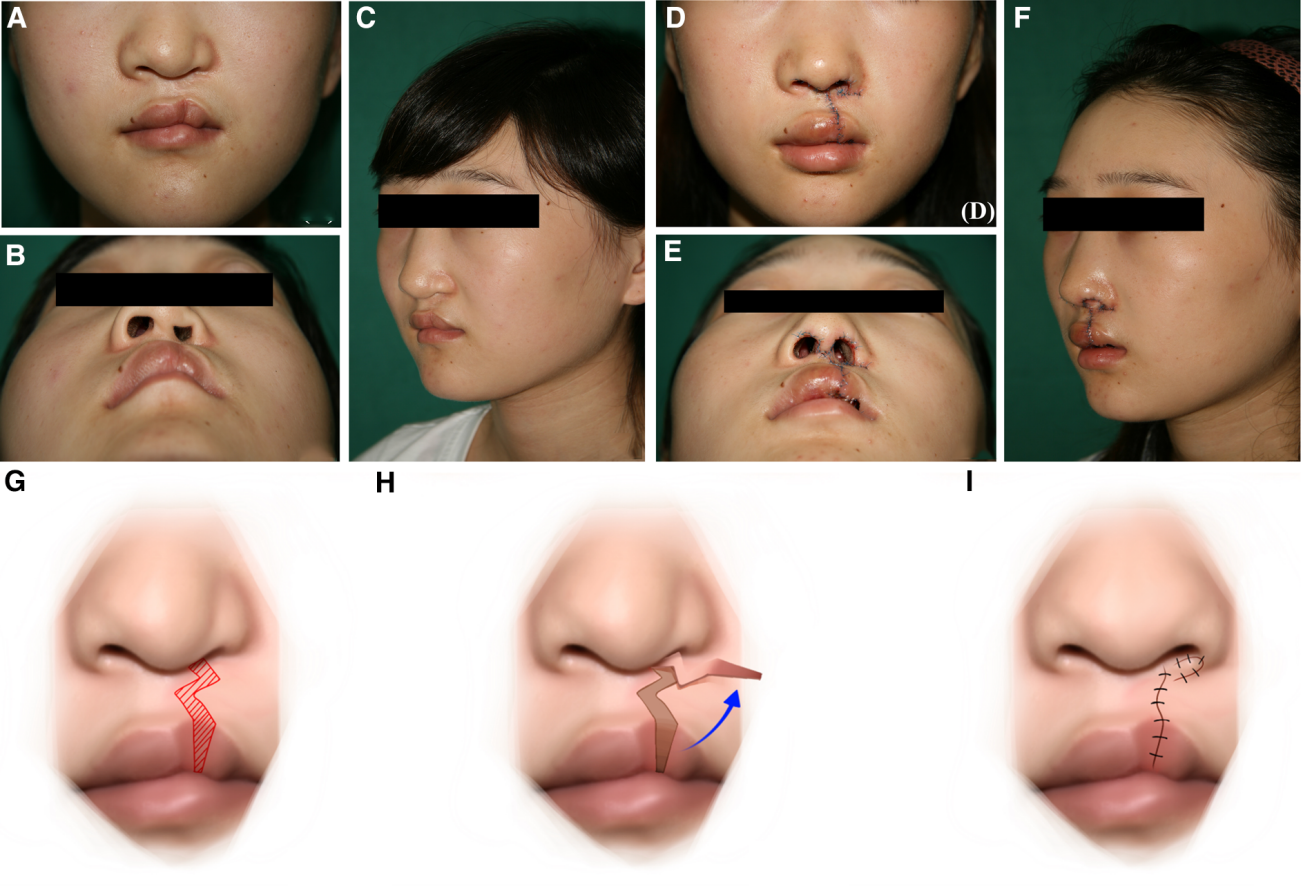
Clinical Presentation 1: A 17-year-old female patient with a narrow nostril secondary to cleft lip and palate corrected by the upper lip scar tissue flap. (**A**) Preoperative frontal view of the patient. (**B**) Preoperative bottom view of the patient. (**C**) Preoperative profile view of the patient. (**D**) Postoperative frontal view of the patient. (**E**) Postoperative bottom view of the patient. (**F**) Postoperative profile view of the patient. (**G–I**) Schematic presentation of the upper lip scar tissue flap.

### 3.2. Clinical Presentation 2

A 20-year-old female patient with a chief complaint of nasal and labial deformities was presented to correct the deformities in our department. This patient received cheiloplasty at the age of 4 months at a local hospital. As the child grew, the nasal and labial tissue grew and changed and the deformity become more apparent. The patient was referred to our department for the urgent need to revise the nasal and labial deformities. The physical examination revealed a wide postoperative scar on the right upper lip. The vermilion-cutaneous junction was disrupted. The right nasal alar was malpositioned downward, and the nasal nostril was asymmetric with a narrow right nostril. The width of the nasal floor and the length of the alar rim were measured to determine the causes of the narrow nostril. In this patient, the main cause of the narrow nostril was a narrow nasal floor. A nasolabial skin flap was designed, transposed, and transplanted to repair the narrow nasal floor of the affected side. Additionally, open rhinoplasty was applied to reposition the nasal alar cartilage and nostril retainer was utilized to maintain the position and shape of the nasal alar. Thus, resection of the residual scar, anatomical reduction of the nasal alar cartilage, repositioning of key anatomic landmarks, and leveling of vertical lip lengths were achieved at the same time during the surgery. In this case, the upper lip scar was an irregular polyline, so the upper lip scar could not be applied to enlarge the nostril. Thus, a nasolabial skin flap was designed and applied to enlarge the nostril on the affected side. Similarly, the nasal alar of the affect side was lateral malposition. To solve this problem, the bilateral tajima incision and the inverted V-shaped incision on the nasal columella was utilized in the surgery to dissociate and reposition the nasal alar cartilage anatomically. The nostril retainer was also applied to maintain the nostril size and alar cartilage position after the surgery for 6 months. A good nasolabial appearance was achieved after surgery. And the photos before and after the secondary rhinocheiloplasty are shown in Figure 2.

**Figure 2:**
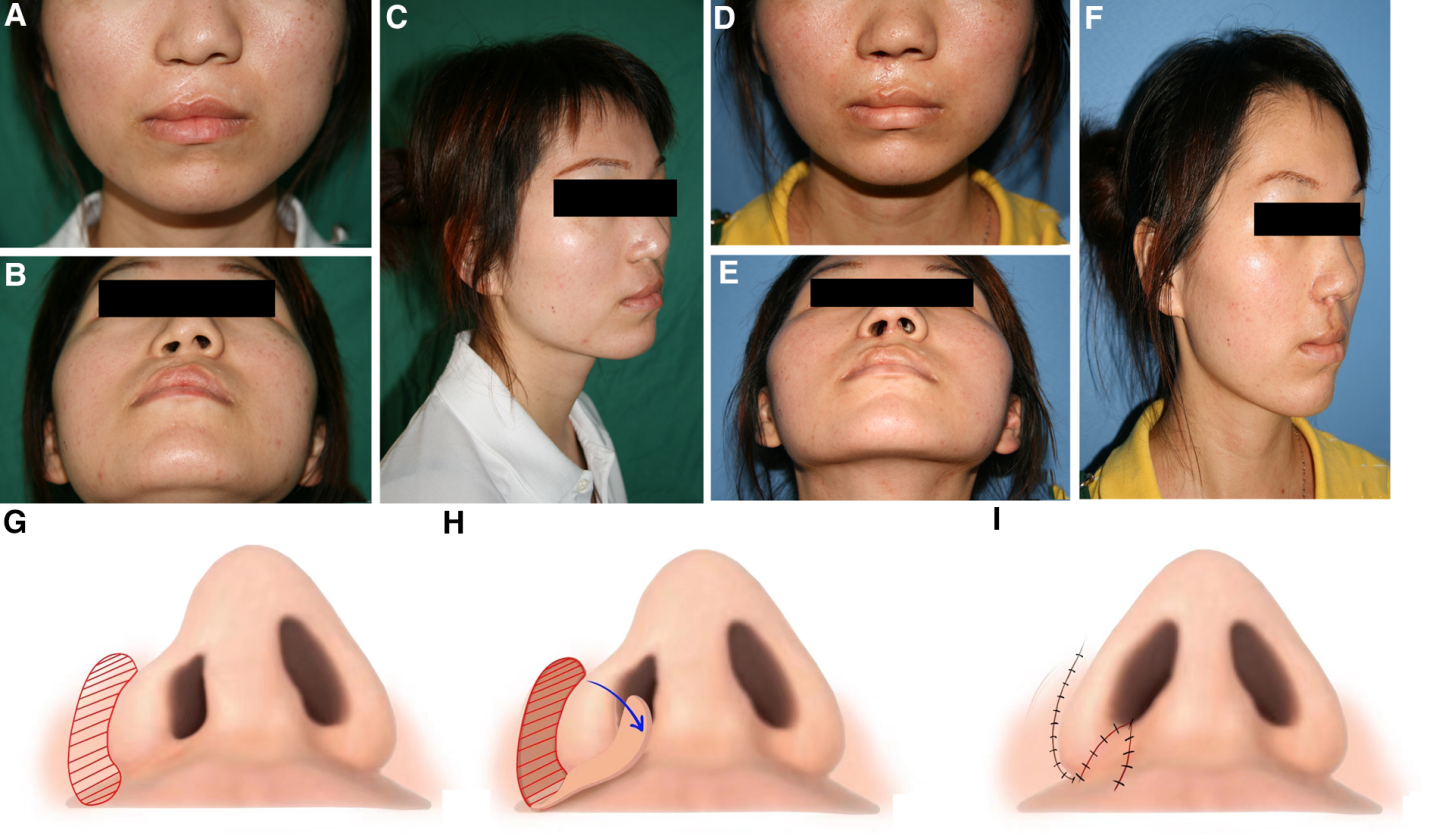
Clinical presentation 2: A 20-year-old female patient with a narrow nostril secondary to cleft lip and palate corrected by a nasolabial skin flap. (**A**) Preoperative frontal view of the patient. (**B**) Preoperative bottom view of the patient. (**C**) Preoperative profile view of the patient. (**D**) Frontal view of the patient 1 month after surgery. (**E**) Bottom view of this patient 1 month after surgery. (**F**) Profile view of this patient 1 month after surgery. (**G–I**) Schematic presentation of the nasolabial skin flap.

### 3.3. Clinical presentation 3

A 22-year-old female patient was diagnosed with right nasal and labial deformities secondary to CLP and presented at our hospital to resolve the narrow nostril of the affected side. This patient underwent cheiloplasty at the age of 3 months and palatoplasty at the age of 10 years at a local hospital. However, a palatal fistula remained after the surgery. The patient underwent surgery to repair the palatal fistula in our hospital at the age of 16 years. The nasal and labial deformities become more apparent with growth and development, and the patient was referred to our department for revision of these deformities. The physical examination revealed collapse of the nasal tip, asymmetry of the nostrils, and deviation of the septum to the left side. The asymmetry of the bilateral nostrils was particularly evident from the bottom view. Before surgery, the length of the alar rim and the width of the nasal floor were measured to establish a reasonable treatment plan. The alar rim on the noncleft side was 38 mm, and the alar rim on the cleft side was only 22 mm. Thus, a huge difference existed between the bilateral perimeters of the alar rims. A free alar composite tissue flap from the noncleft side was used to extend the perimeter of the cleft side alar rim. During the surgery, a wedge-shaped flap was designed and resected from the lateral one-third of the alar rim on the noncleft side. Subsequently, the resected wedge-shaped flap was inserted into the corresponding position on the lateral one-third of the alar rim on the cleft side to lengthen the alar rim on that side. To ensure the survival of the free flap, the nasal alar cartilage was not anatomically repositioned during the primary rhinoplasty. Only the enlargement of the cleft side nostril was performed at this stage. Local compression was performed with vaseline gauze for 14 days. After the primary rhinoplasty, the asymmetry of the nostrils was revised. However, the collapse of the nasal tip and the malposition of the nasal alar cartilage still remained. The revision of nostril asymmetry provided better conditions for further revision of the nasal deformity. One year later, a secondary rhinoplasty with cartilage from the nasal septum was performed. During the secondary rhinoplasty, the bilateral nasal alar cartilage was also freely dissociated and anatomically reduced to remodel the shape of nasal tip and bilateral nasal alar. The bilateral tajima incision and the inverted V-shaped incision on nasal columella was applied this time for anatomic dissection of nasal alar cartilage. A satisfactory aesthetic and functional result was achieved ultimately. The schematic diagram of the surgery is shown in Figure 3.

**Figure 3:**
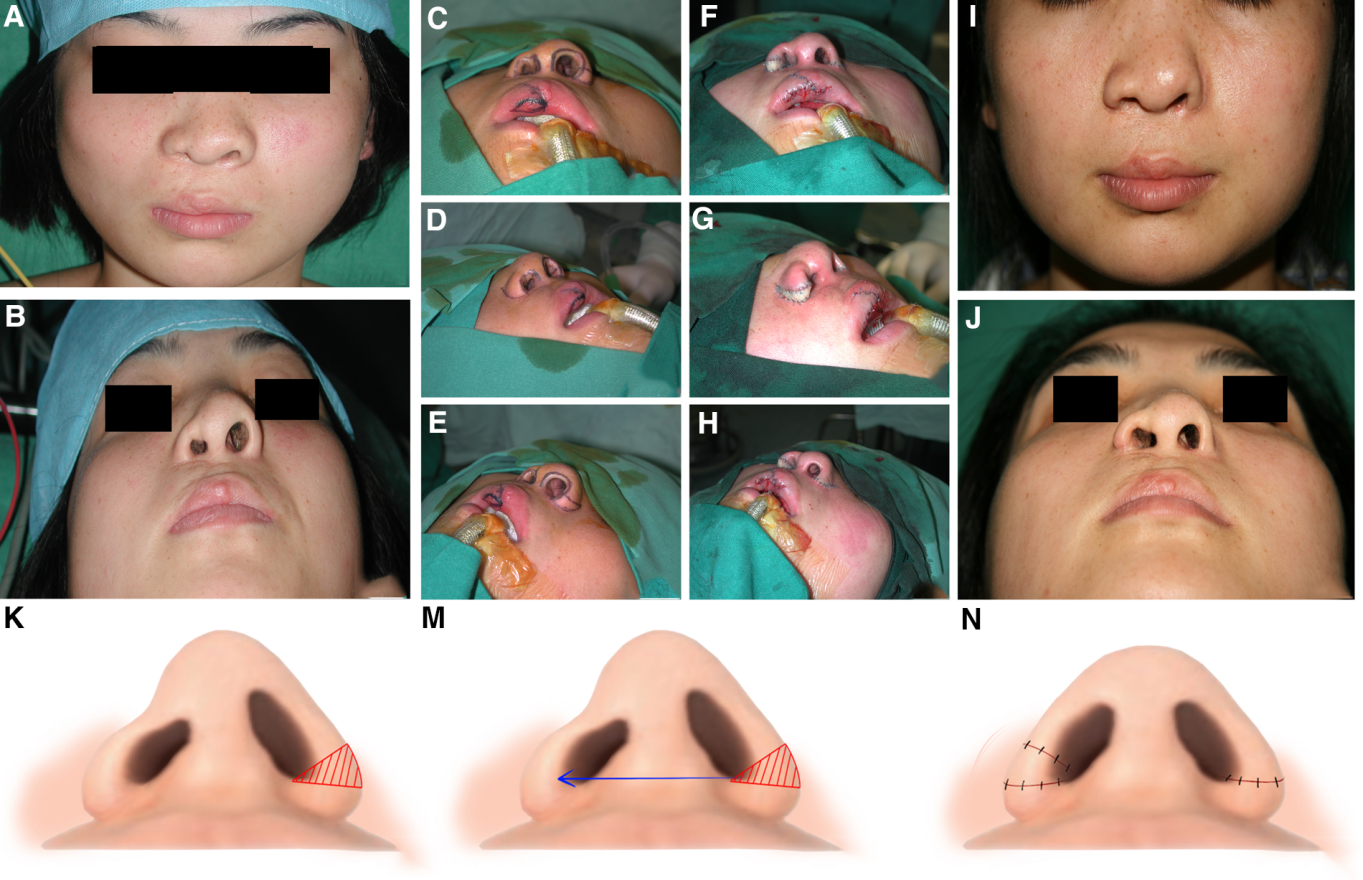
Clinical presentation 3: A 22-year-old female patient with a narrow nostril secondary to cleft lip and palate corrected by a free alar composite tissue flap. (**A**) Preoperative frontal view of the patient. (**B**) Preoperative bottom view of the patient. (**C–H**) Photos during the surgery. A wedge-shaped flap was designed and resected from the lateral one-third of the noncleft side alar rim. This free alar composite tissue flap was inserted into the corresponding position of the cleft side alar rim to lengthen the alar rim. (**I**) Frontal view of the patient 1 year after surgery. (**J**) Bottom view of the patient 1 year after surgery. (**K–N**) Schematic presentation of the free alar composite tissue flap.

## 4. Discussion

Nasolabial morphology is the key to facial aesthetics, and the existence of nasolabial deformities and upper lip scars secondary to CLP trigger great distress in human relationships (7). Although congenital clefts are repaired during infancy and childhood, nasal and labial deformities remain in adolescence and even into adulthood. Nasal deformities associated with cleft lip are characterized by a lack of symmetry, alar collapse on the affected side, loss of tip definition, and obtuse nasal labial angles (10–12). These troublesome maxillofacial deformities result not only from the baseline congenital deformities and deformational changes during craniofacial growth, but also from surgical procedure flaws, poor adherence to instructions in childhood, and maxillary deficiency secondary to CLP (6, 8, 13, 14). These deformities involve abnormalities in all layers, including nasal alar cartilage, nasal septal cartilage, skin, subcutaneous tissue, and even the basal bone (15). Thus, cleft rhinoplasty is regarded as a tough and vital reconstructive procedure. Several factors, such as scars from previous surgeries, insufficient soft tissue for tension-free closure, and displaced and deformed cartilage, might contribute to the final poor postsurgical results (16).

Among all these clinical characteristics, nostril asymmetry is the most common nasolabial deformity secondary to CLP and manifests as increased base width and decreased height (9). However, in clinical practice, the cleft side nostril is sometimes narrow due to improper surgery or overcorrection in a previous surgery. This condition is extremely troublesome due to insufficient soft tissue for nostril augmentation. In addition to the soft tissue defect, scars at the base of the nose and columella, the tight skin envelope, and the intrinsic memory of displaced and deformed cartilage might also cause deformity relapse (17). To resolve this troublesome situation, surgeons have tried various methods to revise this deformity and published some relevant case reports. Several surgical methods, including V-Y advancement flap or *Z*-plasty, are effective in correcting this rare deformity (18, 19). Additionally, Matsuya et. al applied a “flaying-bird” incision in the nostril tip to revise the cleft nasal deformity during open rhinoplasty (20). This procedure widens the nostril from the nostril tip but does not significantly improve the width of the nasal base. Essentially speaking, narrow nostrils are soft tissue deficiencies; thus, these methods result in only short-term improvements in the nasal shape. Slight deformities can be revised by local tissue modifications on the affected side. However, recurrence of the deformity is more likely for severe deformities due to postsurgical scar contracture. Therefore, some surgeons utilize composite tissue flap transplantation during the surgical procedure to revise narrow nostril deformities. To widen the alar base width during post-CLP rehabilitation, Balaji used a typical nasolabial flap to correct the narrow alar base deformity and achieve outstanding esthetic and functional outcomes (21). To restore the symmetry of bilateral nostrils, Suh et. al applied full-thickness skin grafts harvested from the postauricular region to widen the stenotic cleft nostril (18). Although skin grafts appeared as red and elevated scars during the early remodeling period, the transplant area eventually became similar to the surrounding tissues. Lee et. al utilized the subalar grafting technique to improve the symmetry of nostrils and achieved striking results in the immediate postsurgical period and beyond 1 year after surgery (22).

In this study, we summarized the algorithm for the selection of surgical methods for treating narrow nostril deformities secondary to CLP based on the literature and our clinical experience (Figure 4). While designing the surgical plan, all clinical data should be collected, including the patient's chief complaint, medical history, and facial photography. In addition, accurate clinical measurements are important in establishing a surgical plan. We used a steel wire to encircle the alar rim and measured the length of the steel wire to precisely determine the length of the alar rim. Besides that, we used castroviejo calipers to precisely measure the width of the nasal floor and the breadth of the vermilion and scar. Based on the clinical data, the causes of narrow nostril deformities can be divided into three main categories, including a narrow nasal floor, a short alar rim, and a combination of both. When the nasal floor is narrow while the alar rim is normal, a nasolabial skin flap or upper lip scar tissue flap is recommended to utilized for enlarging the width of the nasal floor. To be specific, when there is a broad scar on the upper lip, an upper lip scar tissue flap is recommended to applied priority to avoid opening a second surgical area in the nasolabial fold region. If the upper lip scar is slight and does not need to be revised or the upper lip scar was irregular, a nasolabial skin flap is recommended because the resulting wound is more concealed. When the alar rim is slightly shorter than the other side, adjacent tissue flaps, such as *Z*-plasty or a V-Y advancement flap, are recommended. If the alar rim is significantly shorter than the other side while the nasal floor is normal, free composite tissue flap transplantation, such as a free alar tissue flap or a free auricle tissue flap, is recommended. If the patient is unwilling to receive free tissue flap transplantation and the patient's nostril on the noncleft side is too large, a simple partial resection to narrow the noncleft side nasal alar can also result in a harmonious and symmetrical nasal profile. Finally, when a narrow nasal floor coexists with a short alar rim, a combination of various methods is recommended. The selection algorithm of surgical methods for revising narrow nostril deformities secondary to CLP is shown in Figure 4.

**Figure 4:**
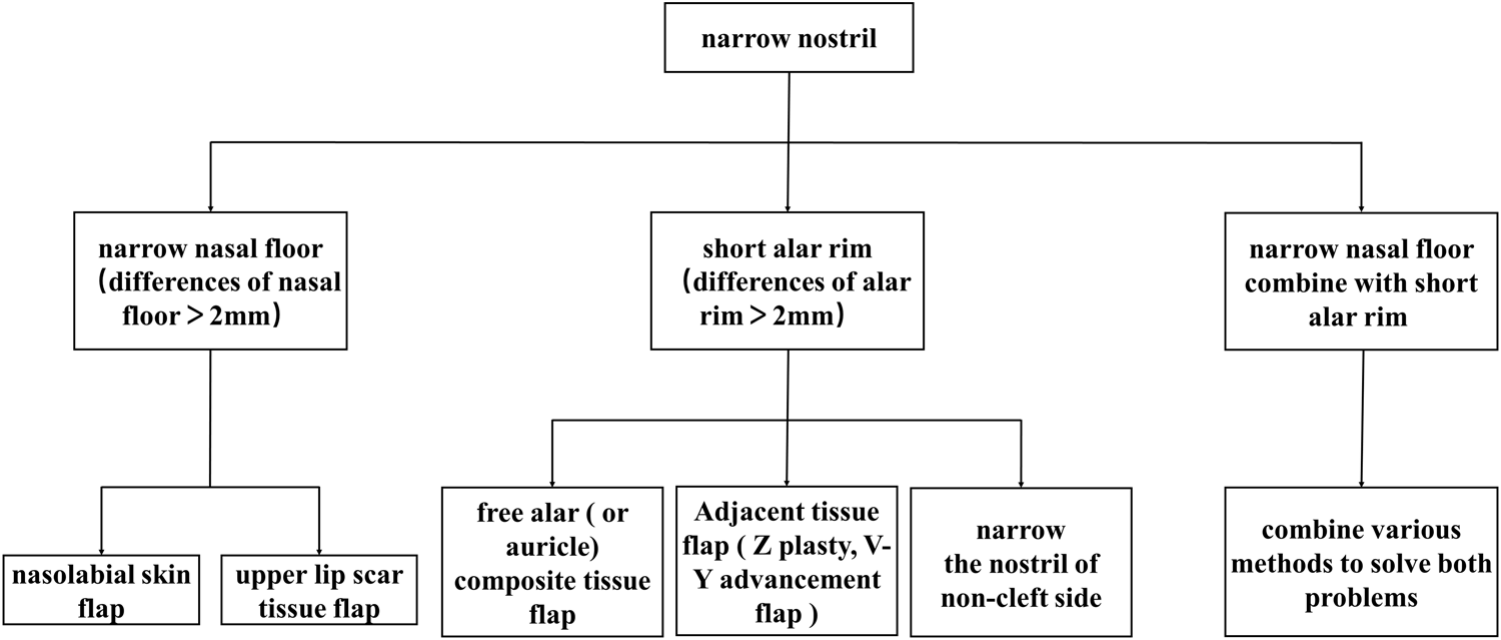
Algorithm for surgical method selection for revising narrow nostril deformities secondary to cleft lip and palate.

Of note, the nostril retainer (Figure 5), which maintains the alar cartilage and the alar wing in the desired positions, is widely applied in surgeries involving the alar cartilage and nostril to prevent alar collapse or nostril stenosis (23–25). However, diversity exists in the literature regarding to the time of postoperative nasal retainer should be applied. Most studies recommend using the nasal retainer for at least six months to maintain the desired surgical outcome (26, 27). However, in our patients, significant recurrence was observed even after the application of the nasal retainer for six months. Severe tissue deficiencies and scar hyperplasia may lead to this phenomenon. Thus, a prolonged application of the nasal retainer is recommended in such patients.

**Figure 5:**
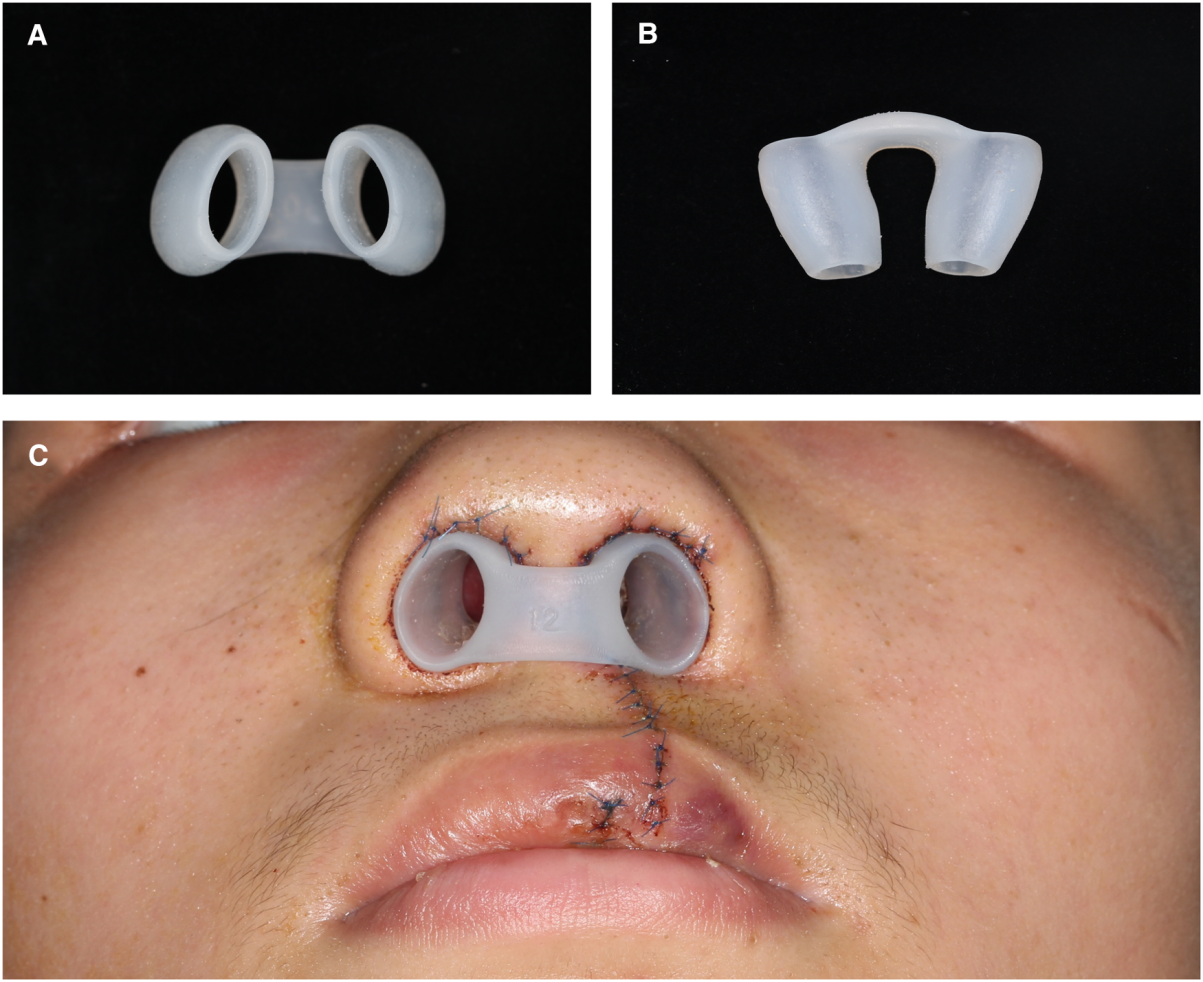
(**A**) and (**B**) the nostril retainer applied after the surgery. (**C**) The nostril retainer placed in bilateral nasal nostril to maintains the alar cartilage and the alar wing in the desired positions.

## 5. Conclusions

Narrow nostril deformities secondary to CLP are rare and troublesome in clinical practice. In this study, we summarized the treatment of these deformities and proposed an algorithm for surgical method selection for revising these deformities. According to this algorithm, the width of the nasal floor and the length of the alar rim are crucial in selecting the correct surgical method. Based on this algorithm, an appropriate surgical method was ascertained and favorable surgical outcomes were achieved.

## Data Availability

The raw data supporting the conclusions of this article will be made available by the authors, without undue reservation.
